# Researcher Perspectives on Data Sharing in Deep Brain Stimulation

**DOI:** 10.3389/fnhum.2020.578687

**Published:** 2020-12-17

**Authors:** Peter Zuk, Clarissa E. Sanchez, Kristin Kostick, Laura Torgerson, Katrina A. Muñoz, Rebecca Hsu, Lavina Kalwani, Demetrio Sierra-Mercado, Jill O. Robinson, Simon Outram, Barbara A. Koenig, Stacey Pereira, Amy L. McGuire, Gabriel Lázaro-Muñoz

**Affiliations:** ^1^Center for Medical Ethics and Health Policy, Baylor College of Medicine, Houston, TX, United States; ^2^Evans School of Public Policy and Governance, University of Washington, Seattle, WA, United States; ^3^Department of Biosciences, Rice University, Houston, TX, United States; ^4^Department of Anatomy and Neurobiology, School of Medicine, University of Puerto Rico, San Juan, Puerto Rico; ^5^Program in Bioethics, University of California, San Francisco, San Francisco, CA, United States

**Keywords:** neuroethics, data sharing, neuromodulation, deep brain stimulation, closed-loop, neural activity data, mental health data, commercialization

## Abstract

The expansion of research on deep brain stimulation (DBS) and adaptive DBS (aDBS) raises important neuroethics and policy questions related to data sharing. However, there has been little empirical research on the perspectives of experts developing these technologies. We conducted semi-structured, open-ended interviews with aDBS researchers regarding their data sharing practices and their perspectives on ethical and policy issues related to sharing. Researchers expressed support for and a commitment to sharing, with most saying that they were either sharing their data or would share in the future and that doing so was important for advancing the field. However, those who are sharing reported a variety of sharing partners, suggesting heterogeneity in sharing practices and lack of the broad sharing that would reflect principles of open science. Researchers described several concerns and barriers related to sharing, including privacy and confidentiality, the usability of shared data by others, ownership and control of data (including potential commercialization), and limited resources for sharing. They also suggested potential solutions to these challenges, including additional safeguards to address privacy issues, standardization and transparency in analysis to address issues of data usability, professional norms and heightened cooperation to address issues of ownership and control, and streamlining of data transmission to address resource limitations. Researchers also offered a range of views on the sensitivity of neural activity data (NAD) and data related to mental health in the context of sharing. These findings are an important input to deliberations by researchers, policymakers, neuroethicists, and other stakeholders as they navigate ethics and policy questions related to aDBS research.

## Introduction

Deep brain stimulation (DBS) and adaptive DBS (aDBS) research are ongoing for a variety of movement disorders and psychiatric disorders. There is wide recognition of the importance of data sharing for the advancement of this research (Deeb et al., 2016; Rossi et al., 2016). While some disease-specific DBS registries exist, no central registry has yet emerged housing information on therapeutic outcomes and technical specifications across different conditions for which DBS is used (Lozano et al., 2019). Addressing this disconnect requires understanding the potential benefits and risks of data sharing, barriers to sharing, and potential solutions to these barriers from the perspective of key stakeholders. Further, a sustainable approach to data sharing must take into consideration DBS researchers’ conceptual understandings and ethical views about data sharing that are informed by their interactions with patient-participants and by knowledge of the evolving scientific details of DBS systems (Lázaro-Muñoz et al., 2019).

## Materials and Methods

We conducted semi-structured, open-ended interviews in which researcher-participants (*n* = 23) were asked about their perspectives on data sharing practices, ethics, and policy in aDBS research. Queried topics included whether, how, and where the researchers we interviewed are sharing their research data, their attitudes toward data sharing from aDBS research, whether they would be uncomfortable sharing any data related to their research, their potential concerns about other researchers having access to their data and how sharing could affect patents or trade secrets, and their attitudes about sharing particular data types [neural activity data (NAD) and data related to mental health]. We developed the interview guide based on a review of key issues and concerns identified in the bioethics and neuroethics literature on data sharing, during participant observation in a lab conducting aDBS research, and in discussions with other experts. While the interview guide included additional questions related to other important neuroethics issues (including what researchers view as the most pressing ethical issues in aDBS research, what issues they have personally encountered in their research, and questions about specific features of aDBS), we report researchers’ views on those topics elsewhere (Muñoz et al., 2020) and report here specifically on results about researchers’ attitudes and perspectives towards data sharing. We have used identification numbers in this piece that are different than those in Muñoz et al. (2020) to help ensure de-identification of researcher-participants.

We conducted 23 interviews, recruiting researcher-participants based on their involvement in aDBS trials. We employed purposeful sampling with a snowball strategy (Patton, 2002; Cresswell and Plano Clark, 2011; Palinkas et al., 2015), including the use of NIH RePORTER. We aimed for the representation of distinct researcher roles (e.g., trial coordinators, neurologists, neurosurgeons, mental health clinicians, and engineers) and target conditions [e.g., Parkinson’s disease, dystonia, essential tremor, Tourette syndrome, obsessive-compulsive disorder (OCD), and depression]. One researcher was involved in conventional DBS and next-generation DBS research but not currently working on aDBS directly. We conducted interviews until reaching theme saturation, defined as the point at which new interviews no longer raised novel themes relative to previous interviews (Saunders et al., 2018). Baylor College of Medicine’s Institutional Review Board approved our research.

Researchers were invited to participate by email. Interviews were conducted *via* phone and Zoom. These interviews were recorded and their transcripts were analyzed with the aid of MAXQDA 2018 qualitative data analysis software (Kuckartz, 2014). Four members of the research team (PZ, KK, LT, and RH) inductively developed a codebook to identify thematic patterns in researchers’ responses to the questions outlined above, as well as in other parts of the interview where researchers discussed their concerns or attitudes about data sharing. Two members of the research team (PZ and CS) applied thematic content analysis (Boyatzis, 1998) to these interview segments to identify a list of more fine-grained themes. These fine-grained themes structure the analysis and frequencies presented below.

## Results

Participant characteristics are summarized in Table 1. The majority of participants were male, white, and had an advanced degree.

**Table 1:** Researcher demographics.

**Gender (*n* = 23)**
Male	13 (57%)
Female	9 (39%)
Prefer not to answer	1 (4%)
**Race/Ethnicity (*n* = 23)**
Asian	3 (13%)
White	18 (78%)
Prefer not to answer	2 (9%)
**What degree(s) do you currently hold? (*n* = 23)**
M.D. or equivalent	8 (35%)
Ph.D. or equivalent (clinical)	3 (13%)
Ph.D. or equivalent (research)	4 (17%)
Both M.D. and Ph.D. or equivalent (clinical)	2 (9%)
Both M.D. and Ph.D. or equivalent (research)	1 (4%)
B.Eng. or M.Sc. Engineering	2 (9%)
B.A. or B.S.	3 (13%)
**Project roles (*n* = 23)**
Clinical trial coordinator	4 (17%)
Engineer	5 (22%)
Mental health clinician	4 (17%)
Neurologist	5 (22%)
Neurosurgeon	5 (22%)
**Research focus (*n* = 23)**
Movement disorders	6 (26%)
Psychiatric disorders	8 (35%)
Both	9 (39%)
**Mean years of research experience (*n* = 23)**
Years of experience related to conventional DBS	8.7
Years of experience related to a DBS	4.5

### Data Sharing Practices and Importance

When asked whether they were sharing data outside their project team, 10 researchers (44%) said that they were sharing at least some data. Another nine (39%) said that they were not currently sharing but planned to in the future. However, among those currently sharing, there was variation in the type of sharing partner, with comparatively few making data available to registries or other research teams (Table 2).

**Table 2:** Data sharing practices.

**Is data currently being shared outside of the**
**project team? (*n* = 23)**
Yes	10 (44%)
No, but planning to do so in the future	9 (39%)
Unsure of project’s data-sharing practices	3 (13%)
Did not express a clear answer	1 (4%)
**If data are being shared, with whom? (*n* = 10)**
Device manufacturers	4 (40%)
Registries	3 (30%)
Other research groups	1 (10%)
Government agency	1 (10%)
Did not specify	1 (10%)

Researchers viewed sharing data as important and provided several reasons why data from aDBS trials in particular should be shared (Table 3). The most common justification for sharing data was to advance aDBS research. Data sharing was seen as particularly important in aDBS research because of the small number of participants in any given trial, making it difficult for individual studies and labs to draw definitive conclusions. As one researcher put it:

“*[T]here aren’t enough people that are implanting these devices for us to move forward because even expert centers are only going to implant a few devices per year every other year. How can you ever get enough data to pull it together? [‥.] So how are we going to collect enough cases to even move the field forward and learn from each other as to what targets, what approaches?” (R_12)*.”

**Table 3:** Importance of data sharing for adaptive deep brain stimulation (aDBS) research.

“**I think the world of aDBS is still small**, so the numbers that are being evaluated at individual sites are small, and **there’s power in the data sharing** to be able to have a broader sense or a broader scope of the disease process to be able to understand it better and to understand the signals better” (R_21).
“I think it’s going to take **large numbers of patients with large numbers of recordings** that are acquired in different settings to really try to get a handle on this
“I think teams, as they’re realizing that science is much more **collaborative and team-based**, I think that’s
“[T]here’s **a lot of other ways that the data could be looked at**. There’s **a lot of other questions** that we’re not even looking at that perhaps could be answered for the same disorder or many others” (R_19).
“[T]he more open and accessible it is, **the more honest the science** is, too, and the more honest everybody is about it. There shouldn’t be anybody feeling like they’re having to hide anything. **It keeps everybody** **working in honest, compliant ways**, I think” (R_04).

Data sharing can also facilitate secondary analysis of one group’s dataset by others. This was seen as important because groups conducting secondary analyses could take up and answer research questions not asked by the original data generators, both in the context of the same disorder and for other disorders. Thus, researchers felt that a collaborative approach to aDBS research is needed. A few researchers also said that data sharing would promote scientific honesty and transparency, which were seen as important commitments in biomedical research.

### Concerns and Barriers Related to Data Sharing

#### Privacy and Confidentiality

Despite recognizing the importance of data sharing to advance research, researchers raised various concerns about sharing data. Nearly all (21, or 91%) researchers mentioned at least one concern, with the most commonly cited concern being participant privacy (mentioned by 15 researchers, or 65%). While most researchers felt that careful de-identification is sufficient to safeguard participant privacy, some suggested that aDBS research has features warranting additional caution (Table 4). The small number of participants in aDBS trials potentially complicates de-identification, and some types of data are more identifying by their very nature (e.g., videos of participants and highly individualized symptoms, such as specific obsessions or compulsions in OCD): “Currently, the number of patients who are enrolled, it’s a small number. With a little bit of identifying information, it might not be that hard to figure who people are. So, I think we need to be thoughtful about making data available” (R_23). Also, a few researchers worried that despite the implementation of privacy protections, there was a potential threat of hacking or data breaches. These researchers were unsure what exactly malicious actors would attempt to do or stand to gain from aDBS data, but counseled caution nonetheless. One researcher explained, “I don’t know what they would do with it, but who knows. The point is we don’t want to find out” (R_19).

**Table 4:** Concerns about participant privacy.

“**We have to be a little careful as to what is identifying and not identifying information** but there aren’t a large number of aDBS studies, For example, […] we have video data of the person, let’s say that’s not made available or some extraction of that is that’s not identifiable. There are so few people in these types of studies that with the data, if someone had all the data we collected in our study, they could probably figure out which person it was, or they might be able to” (R_22).
“[I would feel uncomfortable sharing] data that can be easily tagged to a patient’s identity. That in particular, or certainly anything that has any **financial implication** or whatever. Anything that if lost, could lead to **identity theft**” (R_19).
“**I don’t think videos of the face should ever be shared**. We have to, if we’re sharing the face, a video of someone, I think **their face has to be blurred unless we have their specific consent** to not blur the face” (R_14).

#### Data Usability

A majority of researchers (12, or 52%) also raised concerns about the usability of shared data due to difficulties in interpretation (Table 5). Researchers repeatedly stressed the necessity of including appropriate context and annotation in shared data due to the diversity of measures, collection procedures, and behavioral tasks performed by patients. Without this information, data may be difficult or even impossible to accurately interpret, especially neural data, limiting the usefulness of the data for other researchers. The difficulty of data interpretation could also potentially allow researchers to formally fulfill sharing obligations without the data being genuinely meaningful to others. As one researcher put it, “I’ve had some researchers […] tell me if you want people to not be able to use your data, put it in a registry” (R_12).

**Table 5:** Concerns about usability of shared data.

“[T]he data collected haven’t all been collected the same way, **so they’re not comparable**” (R_06).
“I think generally, **especially with neural data, it’s really hard to interpret it if you weren’t the one collecting it** and you don’t know all the details. So I personally wouldn’t want to use people’s data that I don’t know” (R_14).
**“[I]f data is not annotated very well, it is useless**. If you don’t know exactly when it was collected, how it was collected, what are the various conditions? If those things are not carefully documented, the data’s of limited utility” (R_08).
“I think that often the biggest challenge is that we can each capture whatever data we want to capture at our own sites for the work that we want to do, **but that may not be the same data or may not be captured the same way as other people or other sites**, so then it becomes hard to evaluate those in the same way” (R_21).
“I’m even worried about my students leaving things the way that’s **interpretable** for future students” (R_13).

#### Ownership and Control of Data

A majority of researchers also raised concerns about data sharing related to ownership or control of the data [mentioned by 12 researchers (52%)]. Some researchers felt that because their research is NIH-funded, the data ultimately belongs to society at large and thus ought to be shared. One researcher said, “The data is not really ours. It was paid for by the American taxpayer, so the idea that we can hoard it and not have other people be able to do ethical research on it doesn’t make sense. It belongs to the public, fundamentally” (R_08). All 12 of these researchers were also concerned about control of the data from the perspective of academic or professional fairness. Several worried about sharing data before publishing on it because they did not want to be “scooped” by researchers who did not themselves collect the data. This would be unfair, researchers thought, because of the time and intellectual effort expended designing studies and generating data. They also worried that this could limit career opportunities that depend on receiving appropriate credit for one’s effort. As one researcher summed it up, “Fundamentally, the issue is about recognition for the work that was done to set up this trial and get the data” (R_22).

Researchers (7, or 30%) also raised related concerns about the commercialization of data, expressing concern about how for-profit interests in this data can impact data sharing and progress in the field (Table 6). As one researcher said:

“*[C]ommercialization, in many ways, is the enemy of science. You know, because as soon as you start thinking about commercializing your findings, okay, you want to be careful what you share. And you may also want to be careful about who you include as a collaborator. And you may also want to be careful about the kinds of questions that you ask or measures that you make. I think all of these extra-scientific concerns come in, and they have the potential to really restrict advancement. This has been my experience, and I don’t claim it’s representative, but that these are things that I’ve seen” (R_23)*.”

**Table 6:** Concerns about commercialization of shared data.

“**[A]nything that we can scientifically extract from the brain data, I don’t ethically find that anything out of that should be patented** […] Again, we’re trying to make a scientific contribution. I don’t think a discovery should be patented. A system that’s invented could be, **but I really don’t think scientific discovery should be patented**” (R_13).
“[T]here are other companies in this space who are making their business models off of a **cross-site or cross-disease, cross-study data mining** […] using large, large, large datasets across lots of studies and sites to make insights and it’s interesting that—it seems like **whatever NIH suggests should not be something that is related to a commercial interest**. But I don’t think that’s been made clear from the NIH. I don’t know where they want us to put stuff, if they have a place” (R_22).
“**[A] lot of times we’re the ones developing it, giving them the information they need to take it to the next step, we have the ideas** but we can’t manufacture these devices to put in humans so then they end up doing it and claiming all the IP. **And they end up with the big payday**. So I think we do get screwed at some point on this” (R_12).
“[W]e’re sort of put in a spot where if we want to do this research, we have to use these devices that are coming only from say these companies and so we’re sort of in a bind where we have very little leverage to make any more beneficial arrangement with the company. **We have no leverage in the relationship essentially**” (R_22).

Two questioned the fairness of device manufacturers’ practices related to intellectual property resulting from aDBS research, maintaining that researchers often do not receive benefits commensurate with their vital role in generating these companies’ profits. One of these also offered broader worries about the commercial use of aDBS data, remarking that some companies are engaging in “cross-study data mining” and suggested that NIH should avoid sharing requirements that promote commercial activities of this kind. Other researchers raised concerns about unintended commercial uses of shared data, including for predictive diagnostics, neuromarketing, and neuroenhancement.

#### Limited Resources for Data Sharing

Three researchers (13%) mentioned that resources needed for sharing can be a barrier, particularly time, effort, and funding. One suggested that effective data sharing would require a dedicated research assistant. Another described the difficulty of securely transferring data on a large scale and explained that, in some of their work, physical storage devices were transported between study sites instead: “[R]ight now it can be quite cumbersome to encrypt and transmit large quantities of data. I think for some of the work that we’re doing, actually physical drives have to be sent back and forth because it’s too time-consuming to send electronically” (R_16).

### Potential Solutions to Sharing Challenges

Researchers discussed various solutions to several of these issues (Table 7). Regarding concerns about privacy, researchers suggested safeguards such as facilitation of data encryption and additional protections or tiered access for sensitive data types such as data related to mental health. Researchers felt that data standardization (e.g., implementing a common set of measures and using a common interface or format for sharing data), as well as transparency in analysis techniques, could help manage concerns related to interpretation. In response to concerns about data ownership, control, and professional fairness, researchers suggested professional norms such as a holding period during which researchers would have a reasonable amount of time to publish on their data before sharing, as well as the clear linkage of datasets with the generating researchers and identification of ways to credit these researchers to provide professional incentives. Regarding intellectual property, one researcher suggested that investigators who contribute to improving aDBS devices could potentially share patents with device manufacturers. To address issues related to insufficient resources, it was suggested that streamlining secure transmission of data would ease the burden of sharing. Finally, some described general governance solutions related to the question of sharing practices and policy, mainly looking to NIH for guidance.

**Table 7:** Potential solutions to various types of concerns.

*Privacy*
“I think the major consideration is how to be able to do that in a way that maintains privacy, yeah, just maintaining the privacy and **keeping it within an approved set of investigators, perhaps somehow some approval process or some application process**
*Interpretation*
“I think as repositories get set up, knowing the conditions of the collections and things, and **standardizing that so you know what you’re getting** so that you don’t get an overinterpretation of data is going to be super important” (R_12).
*Ownership*
“I’d like to see a little bit of **a holding period, just for the people who collected it to be able to look at it**. Those are the people who know it best. But beyond some reasonable holding period for those investigators, then it should be shared” (R_08).
*Commercialization*
“If we maybe see an improvement on something, we could **discuss that with [the device manufacturer], and it could be patented**, co-patented, or something like that” (R_13).
*Lack of Resources*
“I think **developing or facilitating the secure transfer of information**” (R_16).

### Sensitivity of Neural Activity Data (NAD)

Several researchers (9, or 39%) commented that NAD is **less sensitive** than other data that are typically shared, such as genetic data. Among the reasons given were: (1) NAD is not inherently identifying (at least at present); (2) NAD does not support inferences about current or future disease state; (3) there is a general lack of knowledge about what information can be gleaned from NAD; (4) it presents a lower risk of stigma and discrimination; and (5) NAD is less informative and definitive than genetic data due to more noise and weaker correlations with phenotypes.

Other researchers (5, or 22%) felt that NAD and genetic data are **equally sensitive** in the context of sharing. These researchers believe that NAD might one day be identifying and could potentially be used in harmful ways, for example, “in a legal case” or “by a health insurance company” (R_04), or for “fingerprinting or identifying somebody” (R_19). One researcher said that NAD could someday affect a person’s life prospects in various areas in a way similar to “HIV status or gene mutation data” (R_08).

Three researchers (13%) felt that NAD is, in fact, **more sensitive** than genetic data because the moment-to-moment mental states it potentially allows to be inferred change over time in a way that one’s genetic makeup does not, and that the gap between genotype and phenotype present in the genetics context is absent in the NAD context. One researcher put it this way: “Your neurological data, it is happening. That is the full expression of what’s going in some part of your body. It may necessarily be more personal because of that” (R_05).

The remaining researchers took more ambivalent positions, with one saying that NAD is **equally or more sensitive** or at least will be once it is better understood, one saying that NAD **might** **be more sensitive** but ultimately being unsure, three saying it was **unclear** whether one is more sensitive than the other, and one not expressing a clear view on the topic.

Several researchers provided specific comparisons and analogies to illustrate their views on the sensitivity of NAD in the context of sharing (Table 8).

**Table 8:** Comparisons and analogies regarding sensitivity of neural activity data (NAD).

**In favor of special sensitivity**
“**15 years from now it could be identical to having patients’ intimate medical records revealed**
“So, I mean, it could be another way of kind of **fingerprinting or identifying somebody** like genetic data, so I think we need to be careful with that” (R_19).
“I think the brain signals should be treated somewhat differently, because they will, if not now then the future, they will be in **this sensitive category like HIV status or gene mutation data** that can affect, in principle, things like someone’s insurability, their job hiring, their compatibility for a partner in life. It potentially will have that same level of sensitivity” (R_08).
“I guess if somebody were to get ahold of that data and use it in some way to discriminate against that patient or exclude them for any reason based on that data, **much like HIV information on patients is protected** because it used to be used against patients by their employers. We certainly wouldn’t want that to happen” (R_04).
**Against special sensitivity**
“**It’s not like DNA. It’s not like an iris scan**. I think if we find a useful biomarker, it’s gonna be relatively universal. That’s what you hope for, right? You’re not looking for something, a neural recording that just identifies that individual. You’re gonna want **something that is common across individuals** for it to be useful” (R_06).
“Again, from a neurophysiology standpoint, **I’m not aware of any fingerprint type situation that could identify a patient**, so I think the concerns are maybe a little less. I think it’s when it gets to other features of that data in terms of either outcomes or symptoms or things of that nature where some of those concerns would come in” (R_21).
“Again, this information in my opinion and I’m not a neurophysiologist, but **I think is very different than for example, DNA, which is much more personal, and potentially identifiable** for a specific individual. Brain recordings are not” (R_17).

### Sensitivity of Mental Health Data

Researchers also offered various views on whether data related to mental health is more sensitive and should be treated differently than other data types in the context of data sharing (Table 9). Several (9, or 39%) maintained that it should be treated differently because data sharing can exacerbate mental health stigma and the risk of discrimination and mental health symptom states are potentially more personal, or least may be *perceived* as revealing more about a person, than other types of data. However, a majority of researchers (12, or 52%) maintained that mental health data should not be treated differently than other data types because data sharing procedures that ensure successful de-identification are not likely to put patient privacy in jeopardy, and because sharing information about mental health conditions is not fundamentally different than sharing information about neurological or other physical illness. These researchers referred to distinctions between mental health and other kinds of conditions as “artificial” (R_19) or “arbitrary” (R_02, R_19) and argued against making such distinctions on scientific and conceptual grounds. One mentioned that drawing a distinction could perpetuate mental health stigma, saying, “I think that we’ve got to get rid of the stigma, and as long as we keep treating it differently, we’re not going to get rid of the stigma that exists […] You don’t do it with other illnesses because it’s not beneficial, and I also think it creates more stigma in our community, you know?” (R_11). Two researchers did not express a clear view on whether mental health data should be treated differently.

**Table 9:** Whether mental health data should be treated differently than other types of shared data.

**Mental health data should be treated differently**
“I think patients’ psychiatric illness and mental health data, **it’s a topic very similar to things like HIV** or other illnesses where patients’ ability to have fulfilling and productive lives would be affected by the release of that information” (R_16).
“[S]omething like EKG data is not as consequential to the patient if it is discovered, whereas mental health, there is **a lot of assumptions that are made** about how a person thinks, and how they act, and what they do. There are a lot more consequences with that data getting released” (R_04).
“A medical diagnosis, such as a depression, or anxiety, or a bipolar disorder, things like that. I think there is some extra sensitivity needed there just because of the **social stigma** of having some of those conditions” (R_01).
“Yes, because of the stigma involved and—yeah, mostly because of stigma, and you worry about how it will **impact relationships and work opportunities**” (R_06).
“I struggle with this. On the one hand, it’s like, ‘Well, no. It’s all health. Let’s stop dividing it.’ But nonetheless, **there is still stigma out there**. There is patient provider. So, I think because of that, we want to consider them essentially separate. One day I hope that answer is, ‘No, it’s all health”’ (R_15).
**Mental health data data should not be treated differently**
“I don’t think so. I mean, we usually do, but **I think it’s a bit artificial**. I mean, mental health diagnoses in my mind are the same as neurological diagnoses and somehow don’t fall in the same category as, I don’t know, further protected data” (R_19).
“**There are people who separate the mind and the body in ways that I feel are arbitrary**. So if you have a mental health illness, it’s really, in my view, a physical illness. It’s a problem with your body, it’s the part of your body that’s located in the brain” (R_02).
“I would hope that we would be thinking about overall you know, human health now and not separating out mental health from the rest. But others would probably find that to be more sensitive than me, I don’t know. **I try not to think about it in any different way than I would heart disease**” (R_09).
“I mean, I think psychiatric and neurological conditions are just different names for the problems in different systems of the same organs. So, **it seems arbitrary to me** to call something a mental health problem and something else a neurological problem and then say that one is more protected or sensitive than the other” (R_19).
“I think we need to treat our brain as we treat **every other organ of our body**” (R_22).

There was some, but not complete, overlap between those who said mental health data is especially sensitive and those who said that NAD is especially sensitive. Of those who said that at least one of the two data-types is sensitive (*n* = 13), five said this about both data-types, four said this about mental health data but not about NAD, and four said this about NAD but not about mental health data.

## Discussion

We conducted interviews with aDBS researchers to learn about their data sharing practices and views on barriers and concerns related to sharing aDBS research data. Most researchers were committed to sharing but were not currently sharing as widely as their expressed commitment might suggest. Researchers expressed several concerns related to data sharing, including concerns about the privacy and confidentiality of participants, usability of shared data by others, ownership and control of data, and limited resources for data sharing, as well as potential solutions to these challenges. We also found that researchers were relatively split on the issues of whether NAD is especially sensitive in the context of sharing and whether data related to mental health should be treated differently from other data types.

These results overlap with themes also identified in work on the attitudes of brain-computer interface (BCI) researchers (Naufel and Klein, 2020). While their work focused on ownership and other rights over neural data, especially on the part of patients, they identified researcher concerns about the following issues: interpretability or meaningfulness of neural data, permitting patients to donate or sell neural data to corporations or other entities, being “scooped,” intellectual property, and resources required to share neural data with patients. They also asked BCI researchers whether raw neural data counts as *medical* data, such that it “contains within it potentially sensitive health information,” with a majority saying that it does (Naufel and Klein, 2020, p. 6). Whereas Naufel and Klein (2020) focus on the sharing of data with patients and patients’ rights over such data, we asked researchers about data sharing more generally and received responses primarily concerning sharing among expert stakeholders (such as other investigators and device manufacturers). They also focus on the sharing of neural data in particular, while our project both asked about aDBS-related data in general and posed specific questions about NAD and mental health data.

Researchers expressed a commitment to sharing, saying that they either already were sharing some data or planned to in the future. However, there was diversity in the extent of sharing, both in terms of data types shared and how widely data was shared. This suggests that more detailed policy guidance may be needed as the field matures. Researchers are likely to support the overarching aims of such policy guidance because they believed that sharing is beneficial and even necessary to advancing scientific discovery related to aDBS due to features of the field such as small sample sizes in most studies. They expressed support in particular for what is plausibly categorized as a *collaborative* or *team science* approach (Little et al., 2017). Such approaches have been employed successfully in the genomics context by the Psychiatric Genomics Consortium (Sullivan et al., 2018) and in neuroimaging by the SchizConnect initiative (Ambite et al., 2015), as well as the adoption of the Brain Imaging Data Structure (BIDS) standard (Gorgolewski et al., 2016) which is now being applied to intracranial electroencephalography data (Holdgraf et al., 2019). The genomics and neuroimaging contexts are therefore likely to offer important lessons that will ideally be transferable to the context of next-generation DBS research.

While researchers were generally optimistic about and supportive of data sharing as a way to promote advancement in the field, they also suggested that the full benefits of data sharing are not being realized. Technical barriers to maximally useful sharing include disparate measures and data formats, as well as lack of annotation that sufficiently contextualizes data for use by others. While these are important challenges that will need to be overcome, they are not different in kind than similar challenges that have been identified and adequately addressed in other research contexts. In genomics research, for example, there are lessons to be drawn from the eMERGE Consortium, such as the use of a coordination center to manage data flow (McGuire et al., 2011). In neuroimaging research, sophisticated annotation tools are available to help promote standardization (Poline et al., 2012). The FAIR principles (**f**indability, **a**ccessibility, **i**nteroperability, and **r**eusability) also offer general but useful guidance for the management and stewardship of scientific data (Wilkinson et al., 2016; FORCE 11, 2020). Lessons from other contexts will of course need to be tailored in such a way as to be responsive to specific features of the aDBS context. Features such as the small number of current research participants, reliance on video data that may include participants’ faces (Girard et al., 2015; Provenza et al., 2019), and the sometimes highly specific nature of symptoms in disorders such as OCD and Tourette syndrome present additional privacy challenges that may make de-identification and aggregation more difficult. Nonetheless, researchers should share data to maximize social benefit and minimize risk to individual participants, and the small number of current research participants arguably strengthens this obligation.

Researcher-participants also raised important questions about what scientific investigators are properly entitled to for having generated these datasets, on the one hand, and what society is properly entitled to for having provided resources such as funding, on the other. These two interests have long been widely recognized as important ethical values in science. The U.N. Declaration of Human Rights affirms rights both “to share in scientific advancement and its benefits” and “to the protection of the moral and material interests resulting from any scientific… production of which [a person] is the author” (United Nations General Assembly, 1948). Researchers’ perceptions regarding academic and professional fairness, as well as obligations to the public, are similar to those historically expressed in the field of genomics during the Human Genome Project. These concerns were met with solutions similar to what our researcher-participants proposed, notably with the implementation of the Fort Lauderdale Agreement and acceptance of the Bermuda Principles, which provided both rapid access to data and publication priority for researchers who generated a given dataset (Kaye et al., 2009; Contreras, 2011). The Bermuda Principles allowed for the achievement of rapid sharing in the spirit of open science while remaining flexible and responsive to the needs of the scientific community (Jones et al., 2018).

The NIH Genomic Data Sharing Policy includes similar practices related to publication priority and seeks to ensure appropriate credit for data generators (National Institutes of Health, 2014). Policymakers in the aDBS research context would do well to attempt to replicate this model, which shows that the interests of society and the interests of scientific investigators may not in fact be in tension when it comes todata sharing.

aDBS research relies on device manufacturers to produce the systems used in these trials. Some researchers raised concerns about the involvement of commercial interests in aDBS research, especially when it leads to what they considered unacceptable uses of data, such as for neuromarketing, cosmetic neuromodulation, and commercially available predictive diagnostics. Strong governance structures are needed to address these concerns and should be informed by frameworks applied in other contexts—for example, Contreras’s (2011) application of the *institutional analysis and development framework* (Ostrom, 1990; Ostrom and Hess, 2006; Madison et al., 2010) to genomic data sharing and Deverka et al.’s (2017) Ostrom-inspired principles for the governance of medical information commons in general.

Likewise, concerns raised about patenting invented systems but not scientific discoveries themselves are reminiscent of controversies involving Myriad Genetics, Inc.’s attempted patenting of BRCA1 and BRCA2 genes (see also Naufel and Klein, 2020, p. 7). In *Association for Molecular Pathology v. Myriad Genetics, Inc.*, the Supreme Court of the United States held that scientific work can be patented only when it “creates something new,” and that “products of nature” therefore cannot be patented (Association for Molecular Pathology v. Myriad Genetics, Inc., 569 U.S. 576, 2013). This decision plainly allows an aDBS device to be patented but might seem to preclude the patenting of brain biomarkers of symptom states discovered in the course of aDBS research. However, strikingly broad method patents have already been obtained for applications of DBS and other methods of neuromodulation, and this overbreadth may in some respects approximate patents on the brain regions themselves (Roskams-Edris et al., 2017). Neuroethics debate on whether such patents are appropriate has already begun (Illes et al., 2019; Kuersten and Wexler, 2019). As a further complication, one might also wonder how courts in the U.S. and elsewhere would respond to arguments that a particular brain biomarker emerges only in response to the *interaction* of a patient’s natural neurophysiological processes with an aDBS device.

In the aDBS context, research depends on a small number of private corporations for devices without which trials could not be conducted. This dependence is potentially problematic, including in the context of data sharing. One researcher directed us to the BRAIN Initiative’s Public-Private Partnership Collaborative Research Agreement template, which includes the following clause regarding what should be included in quarterly progress reports made by BRAIN investigators to device manufacturers:

“*These reports will include all relevant PROJECT DATA. PROJECT DATA refers to all written and otherwise recorded information created or collected in service of the PROJECT PLAN. PROJECT DATA shall include, but are not limited to, raw and analyzed data signals (e.g., electrophysiological recordings) as well as any annotations and interpretations of the data necessary for appropriate analyses and interpretation of such PROJECT DATA (BRAIN Initiative, 2015, 1.6.ii)*.”

Because this is merely a template, it does not necessarily reflect the actual agreements entered into between investigators and device manufacturers. Nonetheless, it is instructive as an expression of a baseline or default norm for BRAIN’s Public-Private partnerships. Data sharing of the kind described may provide device manufacturers with the kind of broad-scope access to data that members of the academic research community currently *lack* concerning one another’s work, particularly if companies are receiving project data from multiple trials. Device manufacturers or other private companies involved in this research may thereby be benefitting from data sharing without similar benefits being made available to the broader community *via* sharing among academic researchers. If so, such a state of play may involve an unfair distribution of benefits and burdens, potentially favoring corporate interests at the expense of research advancement by impeding publicly-funded research from fulfilling an obligation to share benefits with society as a whole. As one possible solution to this sort of issue, stakeholders from the scientific community might consider being more vocal about the kinds of arrangements that they view as ethically preferable to the current state of play, potentially including data sharing by device manufacturers themselves.

Some researchers also believed that particular types of aDBS data raise distinct concerns, ones on which the data sharing experience in other fields does not yield clear lessons. The capacity to record as well as stimulate sets aDBS devices apart even from conventional DBS devices, which are themselves unlike most other implanted devices due to their presence in the brain. Recording capabilities allow for the collection of NAD as a key component of the closed-loop systems these researchers aim to develop. In light of the centrality of various cognitive capacities for prominent theories of personhood (Singer, 1993; Korsgaard, 1996; McMahan, 2002) and recent discussions about how the idea of the brain as the basis of the self applies to issues in DBS in particular (Byram and Reiner, 2014; Mecacci and Haselager, 2014; Racine et al., 2017), a natural question to ask about NAD is whether it may be especially sensitive on this or some other basis. This issue forms part of the broader question of *neuro-exceptionalism*: whether and to what extent neurotechnologies raise special ethical, legal, social, and policy issues (Illes and Racine, 2005; Schick, 2005; Alpert, 2007; Tovino, 2007; Wachbroit, 2008). Scholars have engaged in analogous discussions regarding HIV exceptionalism (Bayer, 1991; Ross, 2001; April, 2010; O’Hara, 2011) and genetic exceptionalism (Rothstein, 2010; Garrison et al., 2019; Martani et al., 2019). Recent commentators have stressed that some types of NAD, such as neural activation patterns related to attention, could be especially sensitive due to the wealth of information they potentially represent for hackers, corporations, and governments (Yuste et al., 2017, p. 161). While advances in data security may mitigate some of these concerns, emerging providers of such security are themselves for-profit corporations, potentially heightening concerns about data commodification (Kellmeyer, 2018, p. 6–7).

The researchers we interviewed were split on the sensitivity of this data, offering apparently competing views about the sensitivity of NAD, including whether it allows for the unique identification of a participant. As we describe in our results, nine researchers believed that NAD was equally or more sensitive than genetic data, nine believed it was less sensitive, four were unsure, and one did not express a view. Naufel and Klein (2020, p. 5–6) found that BCI researchers were also split on the related issue of whether neural data is medical data (thereby at least potentially containing sensitive information about an individual’s health). They report 58% of their participants responding that it is, 22% disagreeing, and 20% holding a “neutral feeling” (*n* = 122).

At least for our participants, however, it is also possible that there is more consensus here than it would initially seem. Some of our respondents who took a neuroexceptionalist view did so because of the anticipated *future*, rather than present, informativeness of data, and some of our respondents who took an anti-neuroexceptionalist view did so because of the *present* lack of informativeness of data. Our results may therefore be partially explained by ostensibly neuroexceptionalist researchers focusing on problematic future uses of data and ostensibly anti-neuroexceptionalist researchers focusing on lack of problematic present uses. On the other hand, other of our respondents appeared to hold in-principle views that do not depend directly on how informative NAD is or even could be. Discerning the true degree of consensus among researchers on data sensitivity concerning current and potential future uses will require further investigation. Such work is pressing, as this issue will only grow in importance with the expansion of aDBS research in particular and research involving neural recording in general, as well as with technological advancements allowing for more efficient integration of data (Hendriks et al., 2019).

Data related to mental health emerged as another potentially sensitive data type. Researchers’ views on whether mental health data should be treated differently in the context of data sharing resembled, in one important respect, scholarly debates about *mental health exceptionalism* (Tovino, 2012; Terry, 2015; Gelpi, 2017). Researchers who said that mental health data should be treated differently overwhelmingly believed that this data is especially sensitive due to stigma and potential discrimination. These researchers described harms of stigma such as the overall negative impact on stigmatized individuals’ lives, unjustified assumptions by others, and potential threats to relationships and work opportunities. Treating mental health data the same as other data types risks overlooking how it may be perceived differently and should thus warrant greater privacy protections to avoid stigma or discrimination. Additional protections and tiered access for mental health data, suggested by some researchers, are broadly in line with recommendations by Dyke et al. (2016).

While a majority of researchers said that mental health data should *not* be treated differently in the context of sharing, it is notable that only one of these researchers explicitly mentioned stigma as a reason for this view. In this respect, our findings cut against recent discussions of mental health exceptionalism in which considerations of stigma often figure in arguments *against* treating mental health differently in addition to arguments *for* doing so. As expressed by one researcher, treating mental health data differently may perpetuate stigma by implying that mental health data is substantively or connotatively different than physical data, revealing a different *type* of illness with potentially worse stigmatization. Further research is necessary to determine whether researchers understood these considerations as implicitly invoking considerations of stigma, or whether they view them as not essentially depending on such considerations. For example, it is possible that researchers see these considerations as being philosophically prior to issues of stigma (believing, e.g., that mental health stigma is unjustified partly *because* there is no scientific or conceptual basis for singling out mental health), and took these considerations as sufficient on their own as a rationale for why mental health data should not be treated differently.

## Limitations

These in-depth interviews were intended to identify the range of responses that researchers would offer when discussing ethical and policy aspects of data sharing. This approach is limited in the sense that it cannot and is not intended to provide generalizable results. In line with established principles of qualitative research, we conducted interviews until reaching theme saturation, understood as a point at which participant-researchers were no longer raising novel themes relative to previous interviewees (Saunders et al., 2018). Doing so allowed us to identify ethical and policy issues for further analysis and gain an understanding of the conceptual and argumentative resources that scientific experts deploy in considering and responding to these issues (Lázaro-Muñoz et al., 2019). Another potential limitation is that our snowball sampling strategy began with a convenience sample and relied on researchers being willing to discuss these issues with us. This recruitment strategy may therefore have limited the range of perspectives encountered. However, we mitigated this by employing NIH RePORTER to identify additional BRAIN-funded researchers conducting work related to aDBS.

## Conclusion

Our researcher-participants offered a rich set of perspectives that are well-positioned to inform ethics and policy analysis of issues related to data sharing in the aDBS research context. These perspectives are crucial for ensuring that normative neuroethics analysis and resultant policy guidance is grounded in an understanding of existing practices and expert knowledge. Some concerns and barriers, particularly those related to privacy, technical issues with the usability of shared data by others, and academic and professional fairness, have parallels in other research contexts. Policymakers and aDBS data generators should consider strategies that have been successful in other research contexts such as the Bermuda Principles and Psychiatric Genomics Consortium’s approach to authorship and appropriate credit, as well as approaches to data standardization in neuroimaging, tailoring these as necessary to the aDBS context. However, researchers also raised distinct issues that existing ethics and policy frameworks useful for other research contexts may require amendment or extension to fully address. One of these is the commercialization of data derived from and utilized by aDBS and other devices utilizing neural recordings. Further empirical neuroethics research is needed to identify the full landscape of commercial involvement in aDBS and other invasive neuromodulation research and assess the ethical and policy implications of such involvement in a way that takes account of the perspectives of all stakeholders, including members of device manufacturing companies. Another issue requiring further empirical neuroethics research is the potential sensitivity of certain data types in the aDBS sharing context. Researchers were relatively split regarding whether NAD and mental health data raise special issues related to sharing. Additional research is needed to better understand the full complexity of aDBS researchers’ views about and justifications for the relative sensitivity of NAD and mental health data. Because NAD and mental health data will increasingly constitute the currency of sharing in the decades to come, it is imperative that potential ethical and policy challenges associated with these data types be anticipated and managed now.

## Data Availability Statement

The datasets presented in this article are not readily available because full datasets must remain unavailable in order to ensure de-identification of interview participants. Requests to access the datasets should be directed to glazaro@bcm.edu.

## Ethics Statement

This study involving human participants was reviewed and approved by Institutional Review Board, Baylor College of Medicine. Written informed consent for participation was not required for this study in accordance with the national legislation and the institutional requirements. Written informed consent was not obtained from the individual(s) for the publication of any potentially identifiable images or data included in this article. Verbal consent was obtained from each research participant before beginning interviews.

## Author Contributions

PZ, SO, LT, GL-M, RH, and DS-M contributed to data acquisition by conducting interviews. PZ, CS, KK, LT, and RH contributed to data analysis. PZ, CS, and GL-M conceptualized the manuscript. PZ drafted the manuscript. KK, BK, AM, and GL-M made substantial revisions to the manuscript. JR, BK, SP, AM, and GL-M served in senior leadership roles for the project. All authors contributed to the article and approved the submitted version.

## Conflict of Interest

The authors declare that the research was conducted in the absence of any commercial or financial relationships that could be construed as a potential conflict of interest.
